# Trapped Between Theological and Medical Notions of Possession: A Case of Possession Trance Disorder With a 3-Year Follow-Up

**DOI:** 10.3389/fpsyt.2022.891859

**Published:** 2022-05-26

**Authors:** Igor J. Pietkiewicz, Urszula Kłosińska, Radosław Tomalski

**Affiliations:** Research Centre for Trauma & Dissociation, SWPS University of Social Sciences and Humanities, Katowice, Poland

**Keywords:** Possession Trance Disorder, dissociation, assessment, religious coping, exorcism

## Abstract

Few studies on Possession Trance Disorder (PTD) describe diagnostic and research procedures in detail. This case study presents the clinical picture of a Caucasian Roman-Catholic woman who had been subjected to exorcisms because of her problems with affect regulation, lack of control over unaccepted sexual impulses, and somatoform symptoms accompanied by alterations in consciousness. It uses interpretative phenomenological analysis to explore meaning attributed by her to “possession” as a folk category and a medical diagnosis; how this affected her help-seeking was also explored. This study shows that receiving a PTD diagnosis can reinforce patients' beliefs about supernatural causation of symptoms and discourage professional treatment. Dilemmas and uncertainties about the diagnostic criteria and validity of this disorder are discussed.

## Introduction

Possession is a broad folk category used to explain a variety of symptoms or problems (1). It is frequently associated with possession-form presentations, marked by: talking in a different voice, sensation of paralysis, shaking, glossolalia or making animal sounds, or “night dances” (2). Members of different religious groups also explain possession as incomprehensible somatic symptoms, difficulties in spiritual practice or even problems in relationships (3–11). Furthermore, people in many groups perceive exposure to inappropriate music or films, using substances, masturbation, homosexuality or extra-marital sex as spiritual threats or indicators of being possessed *per se* (12, 13). Even delusions of possession in patients with schizophrenia spectrum disorders can be ascribed by some priests and community members to demonic possession (14). Although people labeled as “possessed” represent a heterogeneous group in terms of clinical presentations, many anthropologists view possession as an idiom of distress and a way of communicating or expressing protest by those who are marginalized or subordinate (15, 16).

The concept of possession has also been used in psychiatric language (see: Table 1). The 10th and 11th editions of the WHO classifications list Possession and Trance Disorder (PTD) in the dissociative disorders chapter. ICD-11 describes it as: “a marked alteration in the individual's state of consciousness and the individual's customary sense of personal identity is replaced by an external ‘possessing' identity and in which the individual's behaviors or movements are experienced as being controlled by the possessing agent” (17). Symptoms should be involuntary and unwanted, and should not be a part of a collective cultural or religious practice (e.g., Cavadi, deliverance ministries), because suggestible individuals may be prone to exhibit behaviors expected in such situations, especially if they have been exposed to trance states or received teachings about possession. For this reason, possession-form presentations triggered by and occurring only during exorcisms should not qualify for this diagnosis (13). Symptoms should also lead to significant distress or impairment in personal, family, social, educational or occupational functioning (17). However, adopting the role of someone “possessed” can be a source of different gains: primary (abreaction or an opportunity to express conflicting impulses in a culturally acceptable manner) and secondary (attracting the attention of others and evoking respect or awe). According to the ICD, unless these trance episodes are recurrent, a single episode should last at least several days to qualify for this diagnosis. Full or partial amnesia is expected for the trance episode. ICD-11 outlines very superficial boundaries between PTD and Dissociative Identity Disorder (DID) or Partial DID: alternate personality states in PTD are attributed to an external (not internal) possessive agent. This implies there is little difference in the overall clinical presentation of PTD and complex dissociative disorders, except for how patients make meaning of their symptoms.

**Table 1:** References to possession in ICD-10, ICD-11, and DSM-5.

ICD-10	**Possession and trance disorder (F44.3)**
	A. The general criteria for dissociative disorder (F44) must be met: G1. No evidence of a physical disorder that can explain the symptoms that characterize the disorder (but physical disorders may be present that give rise to other symptoms). G2. Convincing associations in time between the symptoms of the disorder and stressful events, problems or needs. B. Either (1) or (2): (1) Trance: Temporary alteration of the state of consciousness, shown by any two of: a. Loss of the usual sense of personal identity. b. Narrowing of awareness of immediate surroundings, or unusually narrow and selective focusing on environmental stimuli. c. Limitation of movements, postures, and speech to repetition of a small repertoire. (2) Possession disorder: Conviction that the individual has been taken over by a spirit, power, deity or other person. C. Both criterion B.1 and B.2 must be unwanted and troublesome, occurring outside or being a prolongation of similar states in religious or other culturally accepted situations. D. Most commonly used exclusion criteria: not occurring at the same time as schizophrenia or related disorders (F20–F29), or mood [affective] disorders with hallucinations or delusions (F30–F39).
ICD-11	**Possession trance disorder (6B63)**
	PTD is characterized by trance states in which there is a marked alteration in the individual's state of consciousness and the individual's customary sense of personal identity is replaced by an external “possessing” identity and in which the individual's behaviors or movements are experienced as being controlled by the possessing agent. Possession trance episodes are recurrent or, if the diagnosis is based on a single episode, the episode has lasted for at least several days. The possession trance state is involuntary and unwanted and is not accepted as a part of a collective cultural or religious practice. The symptoms do not occur exclusively during another dissociative disorder and are not better explained by another mental, behavioral or neurodevelopmental disorder. The symptoms are not due to the direct effects of a substance or medication on the central nervous system, including withdrawal effects, exhaustion, or to hypnagogic or hypnopompic states, and are not due to a disease of the nervous system or a sleep-wake disorder. The symptoms result in significant distress or significant impairment in personal, family, social, educational, occupational or other important areas of functioning.
DSM-5	**Dissociative identity disorder (300.14)**
	A. Disruption of identity characterized by two or more distinct personality states, which may be described in some cultures as an experience of possession. The disruption in identity involves marked discontinuity in sense of self and sense of agency, accompanied by related alterations in affect, behavior, consciousness, memory, perception, cognition, and/or sensory-motor functioning. These signs and symptoms may be observed by others or reported by the individual. B. Recurrent gaps in the recall of everyday events, important personal information, and/or traumatic events that are inconsistent with ordinary forgetting. C. The symptoms cause clinically significant distress or impairment in social, occupational, or other important areas of functioning. D. The disturbance is not a normal part of a broadly accepted cultural or religious practice. Note: In children, the symptoms are not better explained by imaginary playmates or other fantasy play. E. The symptoms are not attributable to the physiological effects of a substance (e.g., blackouts or chaotic behavior during alcohol intoxication) or another medical condition (e.g., complex partial seizures).
DSM-5	**Other specified dissociative disorder (300.15)**
	This category applies to presentations in which symptoms characteristic of a dissociative disorder that cause clinically significant distress or impairment in social, occupational, or other important areas of functioning predominate but do not meet the full criteria for any of the disorders in the dissociative disorders diagnostic class. The other specified dissociative disorder category is used in situations in which the clinician chooses to communicate the specific reason that the presentation does not meet the criteria for any specific dissociative disorder. This is done by recording “other specified dissociative disorder” followed by the specific reason (e.g., “dissociative trance”). Examples of presentations that can be specified using the “other specified” designation include the following: 1. Chronic and recurrent syndromes of mixed dissociative symptoms: This category includes identity disturbance associated with less-than-marked discontinuities in sense of self and agency, or alterations of identity or episodes of possession in an individual who reports no dissociative amnesia. 2. Identity disturbance due to prolonged and intense coercive persuasion: Individuals who have been subjected to intense coercive persuasion (e.g., brainwashing, thought reform, indoctrination while captive, torture, long-term political imprisonment, and recruitment by sects/cults or by terror organizations) may present with prolonged changes in, or conscious questioning of, their identity. 3. Acute dissociative reactions to stressful events: This category is for acute, transient conditions that typically last <1 month, and sometimes only a few hours or days. These conditions are characterized by constriction of consciousness; depersonalization; derealization; perceptual disturbances (e.g., time slowing, macropsia); micro-amnesias; transient stupor; and/or alterations in sensory-motor functioning (e.g., analgesia, paralysis). 4. Dissociative trance: This condition is characterized by an acute narrowing or complete loss of awareness of immediate surroundings that manifests as profound unresponsiveness or insensitivity to environmental stimuli. The unresponsiveness may be accompanied by minor stereotyped behaviors (e.g., finger movements) of which the individual is unaware and/or that he or she cannot control, as well as transient paralysis or loss of consciousness. The dissociative trance is not a normal part of a broadly accepted collective cultural or religious practice.

In the American classification, “possession” was diagnosed as the Atypical Dissociative Disorder diagnosed in DSM-III or DDNOS in DSM-III-R. In DSM-IV (1994), possession and trance were diagnosed as sub-categories of the Dissociative Trance Disorder (DTD), and in DSM-IV-TR they were merged into one, and recognized as a cultural variant of the Dissociative Disorder Not Otherwise Specified [DDNOS, (18)]. In DSM-5 (19), possession-form presentations are linked with criterion A of DID: “Disruption of identity characterized by two or more distinct personality states, which may be described in some cultures as an experience of possession” (p. 292). In the absence of other salient DID features (e.g., amnesia), possession episodes can still be coded under the Other Specified Dissociative Disorder (OSDD) category.

However, only some people with possession-form presentations report clusters of symptoms characteristic of complex dissociative disorders such as DID (10, 20–22). In contrast to that, possession-form presentations in PTD comprise some dissociative symptoms not matching criteria for complex dissociative disorders. Other patients with possession-form presentations are better described in terms of personality disorders, marked by problems with attachment, affect regulation, and internal conflicts associated with aggressive or sexual impulses which they can express in a culturally-legitimate manner (4, 11, 13, 23, 24). These people can be encouraged by community members to attribute unacceptable or shameful impulses to demonic influence. In this way, they can externalize psychological conflicts, reduce feelings of guilt or attract others' attention. According to Pietkiewicz et al. (13) there are qualitative differences between these disowned ego-states and autonomous dissociative parts in complex dissociative disorders, and treating these people as if they had such parts could be iatrogenic. Unfortunately, in patients referred by exorcists for diagnostic assessment of their possession-form presentations this diagnosis is rarely taken into account.

It is intriguing how clinicians' personal beliefs about the phenomenal world (e.g., whether or not invisible entities exist which can influence human behavior) affect clinical judgement and meaning attributed to symptoms. Bayer and Shunaigat (25) postulate, for instance, that ‘real' possession should be differentiated from the nosological spirit possession category. Some authors also mention consulting priests or healers (12, 26), but their role in the diagnostic decision-making was not clear. On the one hand, this might express clinicians' sensitivity to patients' religious beliefs and their expectations to involve spiritual leaders in the treatment plan. On the other hand, it could also reveal clinicians' own doubts about the nature of the symptoms. Inability to explain them in terms of psychological mechanisms responsible for a given problem can lead some professionals to consider non-medical explanations. For example, Khan and Sahni (27) explicitly shared their belief that “exorcists, at times, are able to tell whether a person has a mental illness and requires hospitalization and drug treatment or is truly possessed” (p. 254). In another case study by Hale and Pinninti (26), prison and hospital chaplains concurred on “genuine” possession of a patient leading authors to the following conclusion: “If we are to accept that there is a place for belief in real possession in current thinking, then what we have described above might be construed as a case of exorcism-resistant ghost possession, successfully treated with a depot neuroleptic” (p. 388).

Assuming that guidelines for diagnosing possession episodes and information about their prevalence or risk factors quoted in psychiatric manuals are based on research, we decided to review the procedures which were applied to make diagnoses in 48 studies exploring symptoms of possession and trance (see: Table 2). We included publications previously examined in systematic reviews by During et al. (65) and Hecker et al. (66). These two were the only reviews of PTD/DTD available. We also identified nine additional studies of PTD not included in previous reviews. The majority of research on possession-form presentations consists of case studies describing phenomenological aspects, but offering meager descriptions of participants' clinical presentations which could facilitate differential diagnoses. In their reviews, both During et al. (65) and Hecker et al. (66) also included anthropological publications or ethnographic accounts, and studies in which participants obtained other diagnoses, but they were treated as examples of PTD. In 25 studies, diagnoses were based on general psychiatric examination during which patients reported changes in behavior which they attributed to demonic possession. However, authors provided no information about the scope of these clinical interviews and how differential diagnoses were made. Twenty studies suggested the PTD/DTD diagnosis but there was no diagnostic assessment performed by a mental health professional or they contained no information about assessment whatsoever. In four of these studies, assessment was limited to self-report instruments which cannot be regarded as a satisfactory diagnostic procedure *per se*. In four studies diagnoses were determined retrospectively based on analyzing medical records (32–34, 56). Only in four studies, in-depth clinical interviews were used in the assessment, e.g. the Dissociative Disorder Interview Schedule, DDIS (37, 53) and the Structured Clinical Interviews for DSM–IV (SCID) or parts of it (20, 54).

**Table 2:** Review of studies exploring possession trance.

**No**.	**Study**	**Country**	**Study type**	**No. of participants**	**Aim**	**Diagnostic procedure**	**Diagnosis**	**Diagnostic criteria**
1	Bakhshani et al. (28)^b^	Iran	Cross-sectional study	21 out of 4,129	To describe Djinnati and examine its prevalence and demographic attributes in the rural population of Baluchistan in southeast Iran.	Psychiatric examination Self-report instrument: *Dissociative Experiences Scale (DES)*	DTD and Culture-bound syndrome (*Dijnnati*)	DSM-IV
2	Bayer et al. (29)^b^	Jordan	Descriptive study	179	Describing the clinical features of patients who believed they were possessed or influenced by Jinn.	Psychiatric examination	“Possessive disorder” (*Jinn*)	No data provided
3	Butt et al. (30)	Pakistan	Cross-sectional study	350	To determine the frequency of anxiety and depression among patients with dissociative trance (possession) disorder.	Psychiatric examination Self-report instrument: *Hospital Anxiety and Depression (HAD) - Urdu version*	PTD	ICD-10
4	Castillo et al. (31)^a, b^	South Asia	Case study	2	Reexamining previously published cases of spirit possession from the dissociation theory perspective.	No data provided	“Spirit possession”	No data provided
5	Chand et al. (32)^a, b^	Oman	Retrospective chart review	19 out of 111	Retrospective analysis of clinical manifestations and psychosocial aspects of dissociative disorders.	Psychiatric examination: “Information extracted from case records included demographic variables, illness variables, and psychosocial variables [...] Patients with dissociative trance disorder presented with altered state of consciousness, screaming and irrelevant talk.”	DTD	ICD-10
6	Chaturvedi et al. (33)^b^	India	Retrospective chart review	84 out of 893	To examine patterns of dissociative disorders among subjects attending psychiatric services over a period of 10 years.	Psychiatric examination	PTD	ICD-10
7	Das et al. (34)^a^	India	Retrospective chart review	2–4 out of 42	Comparing the suitability of DSM-III-R and ICD-10 criteria for dissociative states	Psychiatric examination	DDNOS (n=2) or PTD (n=4)	DSM-III-R and ICD-10
8	Dein (35)	UK	Case study	1	To illustrate the relationship between spirit possession and psychiatric treatment in a 42 year-old Catholic women.	Psychiatric examination	“Dissociative trance and possession disorder”	No data provided
9	Delmonte et al. (20)	Brazil	Case study	1	A comprehensive account of possession experiences, associated sensations and social interactions	Clinical interview: *Structured Clinical Interview for DSM-5* (*SCID*)	Ruled out DID (“non-pathological possession”)	DSM-5
10	Etsuko (36)^a^	Japan	Case study	1	Comparing folk and psychiatric interpretations of fox possession.	No diagnostic assessment	“Fox possession”	No data provided
11	Ferracuti and Sacco (37)^a^	Italy	Case series	10	Clinical assessment of people with possession-trance states	Clinical interview: *Dissociative Disorders Interview Schedule (DDIS)*, Psychological tests: *Rorschach, Standard Progressive Matrices (SPM)*	DTD	DSM-IV
12	Ferracuti and DeMarco (38)^a^	USA	Case study	1	Describing a case of a man with DTD who was sentenced for the homicide of a 6-month-old baby girl during satanic ritual.	Psychiatric examination Neuroimaging: *CT, EEG* Psychological tests: *MMPI, WAIS-R, Rorschach, TAT*	DTD and Histrionic-dependent personality disorder	DSM-IV
13	Freed and Freed (39)^a^	India	Ethnographic study	38	Describing traditional ghost beliefs and cases of ghost possession among villagers	No diagnostic assessment	“Ghost possession”	No data provided
14	Gaw et al. (40)^a, b^	China	Case series	20	Describing clinical characteristics of patients who believed they were possessed	No data provided	“Possession states” (*kwei-fu, dzao-mo, zhong-xea)*	Chinese diagnostic criteria
15	Guenedi et al. (41)^b^	Oman	Case study	1	Presenting a case of a man in an altered state of consciousness and comparing its phenomenological features with functional abnormality in specific regions of the brain in order to “link possession to brain abnormality.”	Psychiatric examination Neuroimaging: *CT, EEG, SPECT* Psychological test: *MMSE*	An organic pathology: functional changes in the temporal lobe and structural abnormality in the left basal ganglia	No data provided
16	Hale and Pinninti (26)^a, b^	UK	Case study	1	Presenting pharmacological treatment.	Psychiatric examination	Dissociative state or paranoid schizophrenia “Exorcism-resistant ghost possession, successfully treated with a depot neuroleptic.”	No data provided
17	Hanwella et al. (6)	Sri-Lanka	Case study	3	Presenting three patients from Sri Lanka whose possession states were strongly influenced by different religious beliefs and backgrounds	No data provided	“Possession state” (*n* = 1); “Trance and Possession State” (*n* = 1); Acute Stress Reaction (*n* = 1)	No data provided
18	Igreja et al. (5)^b^	Mozambique	Cross-sectional study	175 out of 941	To evaluate the prevalence of self-reported spirit possession in Mozambique.	Self-report instruments: *Harvard Trauma Questionnaire (HTQ)* Questionnaire about spirit possession experience and health-seeking behavior	“Spirit possession” (two subtypes: possession trance and *ku tekemuka*)	No data provided
19	Khalifa and Hardie (3)^a^	UK	Case study	2	Describing cultural, religious and psychiatric aspects of jinn possession.	No diagnostic assessment	“Jinn possession”	No data provided
20	Khan and Sahni (27)^b^	Nepal	Case study	1	To present a case of possession syndrome in a 20 year-old Hindu girl from Nepal.	Psychiatric examination Assessment by an exorcist	“Possession syndrome”	No data provided
21	Khattri et al. (42)	Nepal	Cross-sectional study	4 out of 66	To find out the prevalence of dissociative convulsions type in psychiatric patients suffering from dissociative disorder.	Psychiatric examination	PTD	ICD-10
22	Khoe and Gudi (43)	China	Case study	1	To demonstrate an atypical presentation of panic disorder which imitated episodes of possession trance.	Psychiatric examination Neuroimaging: *EEG, MRI*	Panic disorder with culture specific symptoms	DSM-5
23	Khoury et al. (44)^b^	Haiti	Ethnographic study	4	To investigate whether explanatory models of mental illness invoking supernatural causation result in care-seeking from folk practitioners and resistance to biomedical treatment.	No diagnostic assessment	“Moderate to severe mental illness”	No data provided
24	Kianpoor and Rhoades (8)^b^	Iran	Case series	10	Presenting psychopathology of Djinnati and discussing it in the light of socio-cultural, communication, and dissociation/psychoanalytic theories.	Psychiatric examination	Culture-bound syndrome (*Djinnati*) and PTD or DTD	ICD-10 and DSM-IV
25	Martinez (23)	Puerto Rico	Case study	1	Presenting a case of a man with possession and glossolalia experiences, the diagnostic and therapeutic process.	Psychiatric examination	DDNOS	DSM-IV
26	Mattoo et al. (45)^a^	India	Case study	10	Describing a case of family hysteria and issues related to its medical and social management.	Psychiatric examination Neuroimaging: *EEG*	PTD and BPD (*n* = 1) “Mass hysteria manifest with possession attacks and dissociative symptoms” (*n* = 9)	ICD-10
27	Mercer (9)^a^	USA	Review study	1 out of 2	Describing the impact of the Protestant belief system on the psychopathology and clinical interventions among children and adolescents raised in that religious context.	No diagnostic assessment	“Trance state”	No data provided
28	Neuner et al. (46)^b^	Uganda	Cross-sectional study	91 out of 1,113	To estimate the frequency of harmful spirit possession phenomena and to evaluate the validity of harmful spirit possession as psychological disorder in the case of Northern Uganda.	Self-report instruments: *Cen Spirit Possession Scale, The Violence, War and Abduction Exposure Scale, Posttraumatic Stress Diagnostic Scale, Hopkins Symptom Checklist (Depression section), Luo Functioning Scale, Mini International Neuropsychiatric Interview (module C for suicide risk), Perceived Stigmatization Questionnaire, Aggression Questionnaire*. Survey about the presence of 12 common complaints or symptoms (e.g., malaria, diarrhea, etc.) in the 4 weeks prior to the screening.	“Spirit possession” (*Cen*)	DSM-IV
29	Ng (47)^b^	Singapore	Case series	55	Describing the characteristic features of trance states in three different ethnic communities (Chinese, Malays and Indians).	Psychiatric examination	DTD	DSM-IV
30	Ng and Chan (48)^a, b^	Singapore	Case-control study	58 out of 116	To study the psychosocial stressors that precipitate DTD and to identify predictors of DTD.	Psychiatric examination: “Consecutive cases seen at the psychiatric hospital diagnosed with DTD were included in the study. The psychiatric diagnosis, assigned on the basis of information obtained in a semi structured psychiatric interview and hospital chart review, were made according to DSM-IV criteria”	DTD	DSM-IV
31	Peltzer (49)^a^	Malawi	Descriptive study	116	Describing the nosology and etiology of Vimbuza experience.	No data provided	“Spirit disorder” (*Vimbuza*)	DSM-III
32	Pereira et al. (50)^a, b^	India	Case study	2	Describing cases of possession by a goddess and an evil spirit.	No data provided	“Spirit possession”	No data provided
33	Piñeros et al. (51)^a^	Colombia	Ethnographic study	9	To describe a collective episode of psychogenic illness in an indigenous group (Embera).	No diagnostic assessment	“Embera” (*Mass hysteria*)	DSM-IV
34	Prakash et al. (52)^a^	India	Case study	1	Describing a woman a with precipitation of possession disorder by treatment with nortriptyline.	Psychiatric examination Neuroimaging: *EEG*	Dissociative epileptic disorder	ICD-10
35	Ross et al. (53)	USA	Cross-sectional study	1 out of 100	To determine the prevalence of classical culture-bound syndromes among psychiatric inpatients with dissociative disorders.	Clinical interviews: *Dissociative Disorders Interview Schedule (DDIS), Dissociative Trance Disorder Interview Schedule (DTDIS)* Self-report instrument: *Dissociative Experiences Scale (DES)*	DTD (*n* = 1) Culture-bound syndromes: latah (*n* =1 1), amok (*n* = 11), bebainan (*n* =2 6), pibloktoq (*n* = 3)	DSM-IV
36	Sar et al. (54)^b^	Turkey	Cross-sectional study	13 out of 628	To determine the prevalence of possession experiences and paranormal phenomena among and their relationships with traumatic stress and dissociation in Turkish women.	Self-report instruments: *Childhood Abuse and Neglect Questionnaire* Clinical interviews: *SCID (PTSD and BPD sections), SCID-PTSD (17-items part)*, *Dissociative Disorders Interview Schedule (DDIS)*	“Possession experiences” and DID (*n* = 2) or DDNOS (*n* = 7) or Depersonalization disorder (*n* = 2) or Dissociative fugue (*n* = 1) or Not diagnosed (*n* = 1)	DSM-IV
37	Satoh et al. (55)^a^	Japan	Case study	1	To illustrate diagnostic difficulties in patient whose possessive state and suicidal thoughts were precipitated by door-to-door sales.	No data provided	(DSM) Brief Reactive Psychosis and DDNOS and Somatization disorder (ICD) Somatization disorder and Acute and Transient Psychotic Disorder and Dissociative Disorder	DSM-IVand ICD-10
38	Saxena and Prasad (56)^a^	India	Retrospective chart review	6 out of 62	Presenting clinical characteristics and subclassification of dissociative disorders in psychiatric outpatients in India.	Psychiatric examination	Possession disorder (subcategory of Atypical Dissociative Disorder)	DSM-III
39	Schaffler et al. (57)	Dominican Republic	Cross-sectional study	47 out of 85	To evaluate demographic variables, somatoform dissociative symptoms, and potentially traumatizing events in the Dominican Republic with a group of Vodou practitioners with or without the experience of spirit possession.	Self-report instruments: *Somatoform Dissociation Questionnaire (SDQ-5), Traumatic Experience Checklist – Dominican Republic (TEC), Spirit Possession Questionnaire – Dominican Republic (SPQ);* Interview-based survey (limited data provided). Participants were classified as ‘possessed' upon their positive answer to a screening question whether or not they had experienced full possession by spirits at least once in their lifetime.	“Spirit possession”	No data provided
40	Schieffelin (58)^a, b^	Papua New Guinea	Ethnographic study	4	Analyzing the Evil Spirit Sickness among the Bosavi people of Papua New Guinea during a period of intense Christian evangelization and religious excitement.	No diagnostic assessment	“Evil Spirit Sickness”	No data provided
41	Sethi and Bhargava (59)^a^	India	Case study	7	A description of possession simultaneously affecting seven family members.	No data provided	“Mass possession state”	No data provided
42	Somasundaram et al. (60)^a, b^	Sri Lanka	Cross-sectional study	90	Describing phenomenology of possession states among psychiatric patients, somatic patients and local mediumship adepts of Tamil society in Northern Sri Lanka.	Psychiatric examination (*n* = 30) No data provided (*n* = 60)	“Possession states” (*n* = 90) Psychiatric sample included patients with: Schizophrenia (*n* = 16), Acute psychotic illnesses (*n* = 6), Bipolar disorder (*n* = 1), Dissociative disorder (*n* = 3), Somatoform disorder (*n* = 4)	ICD-10
43	Somer (61)^a^	Israel	Case series	4	To describe how patients used cultural idioms of spirit possession to describe their suffering.	No data provided	DDNOS / DTD (*n* = 1), PTSD (n=1), Schizophrenia (*n* = 1), Histrionic personality disorder and conversion disorder with seizures (*n* = 1).	DSM-IV
44	Szabo et al. (7)^a, b^	South Africa	Case study	1	Describing a female adolescent with features of DTD as part of recovery from major depression following the death of her father	Psychiatric examination Neuroimaging: *EEG*	DTD and Major depressive disorder	No data provided
45	Trangkasombat et al. (62)^a^	Thailand	Descriptive study	32	To describe epidemiological and clinical aspects of the spirit possession epidemic in Thai girls.	Psychiatric examination corroborated with a family interview. Self-report instruments: *Children's Depression Inventory (CDI)* Examination of medical records.	“Mass hysteria” (*n* = 32) DSM diagnoses: Adjustment disorder (*n* = 9), Dysthymia (*n* = 1), Major depressive disorder (*n* = 2), Anxiety disorder (*n* = 1), Dissociative disorder (*n* = 1), Dissociative tendency (*n* = 1), Histrionic personality trait (n=6).	DSM-IIII-R
46	Van Duijl et al. (24)b	Uganda	Case-control studies	119 out of 190	To explore the relationships between spirit possession, dissociative symptoms and reported potentially traumatizing events in Uganda.	Self-report instruments: *Spirit Possession Questionnaire -Uganda (SPQ), Checklist Dissociative Symptoms for Uganda (CDS), Dissociative Experiences Scale (DES), Somatoform Dissociation Questionnaire (SDQ), Harvard Trauma Questionnaire (HTQ), Traumatic Experiences Checklist (TEC)*	“Spirit possession”	No data provided
47	Witztum et al. (63)^a^	Israel	Case study	1	Describing the treatment of a 24 yr-old man with major depressive disorder who complained about being persecuted by an angel.	Psychiatric examination	Major depressive episode with psychotic features and “Hysterical psychosis”	DSM-III-R
48	Witztum et al. (64)^a^	Israel	Case study	3	To illustrate the Zar phenomenon and discuss its cultural and anthropological aspects.	Psychiatric examination Neuroimaging: EEG, CT	Culture-bound syndrome (*Zar*)	ICD-10 and DSM-IV

a *Studies (n = 28) reviewed by During et al. (65)*.

b *Studies (n = 21) reviewed by Hecker et al. (66)*.

Considering the above, rigorous clinical studies exploring possession-form presentations are paramount. They should go beyond phenomenological descriptions and analyze participants' overall functioning in different areas, symptom dynamics, psychological conflicts and mechanisms, attribution of meaning and potential gains. While diagnostic manuals emphasize patients' meaning-making, few studies explored how patients made sense of being diagnosed with Possession Trance Disorder, and how this affected their help-seeking behavior. This case study describes the clinical presentation of a Catholic woman referred for a diagnostic assessment by priests who grew helpless about her aggressive behavior and acts of vandalism. What meaning she ascribed to her PTD diagnosis and how it affected her help-seeking pathways during a 3-year period until a follow-up interview was also analyzed

## Methods

This study was carried out in Poland between 2016 and 2021. Qualitative data included clinical interviews and psychiatric mental health assessment. Their transcripts were subjected to Interpretative Phenomenological Analysis (IPA), which is grounded in phenomenology, hermeneutics, and idiography (67). IPA explores participants' experiences and interpretations, followed by researchers trying to make meaning and comment on these interpretations. Samples in IPA studies are small, homogenous, and purposefully selected. Qualitative material is analyzed in detail case-by-case (67, 68). IPA was chosen for this case study to explore the help-seeking pathways and meaning attributed to the diagnosis of a Possession Trance Disorder.

### Procedure

This case study is part of a larger project examining phenomena and symptoms reported by people using exorcisms. This project was held at the Research Center for Trauma and Dissociation, financed by the National Science Center Poland, and approved by the Ethical Review Board at SWPS University. Potential candidates enrolled themselves *via* a dedicated website, or were registered by healthcare providers and pastoral counselors. They filled in demographic information and completed online tests, including: Somatoform Dissociation Questionnaire [SDQ-20, (69)], Dissociative Experiences Scale - Revised [DESR, (70)]. (Elevated scores in these tests, SDQ-20 ≥30 and DESR ≥72, are suggestive of dissociative disorders). They were then subjected to semi-structured interviews exploring their biography, family situation, religious socialization and spiritual involvement, and motives for enrolling in the study, followed by a diagnostic consultation using Trauma and Dissociative Symptoms Interview [TADS-I, (71)]. The TADS-I is a semi-structured interview intended to identify DSM-5 and ICD-11 dissociative disorders. It includes a significant section on somatoform dissociative symptoms and a section about other trauma-related symptoms. The TADS-I also explores symptoms indicating a division of the personality and alterations in consciousness. Interview recordings were assessed by three healthcare professionals experienced in the dissociation field, who discussed each case and consensually came up with a diagnosis based on ICD-11. This interview was followed by an additional mental state assessment performed by the third author, who is a psychiatrist. He collected medical data, double-checked the most important symptoms, confirmed and communicated the diagnosis and discussed available coping strategies. All interviews and the medical consultations were divided into 60 min sessions.

Among 23 people who enrolled in the project, 12 had features of a personality disorder, five had a schizophrenia spectrum disorder, two met ICD-11 criteria for partial DID, two had Complex PTSD, one had a Dissociative neurological symptom disorder, and one had Possession Trance Disorder. The person with PTD was selected for this analysis. An additional follow-up interview was performed with her 3 years later to explore the meaning she had attributed to her diagnosis and how it influenced her help-seeking behavior. The total length of all interviews conducted with her was 8 h 46 min.

### The Participant

This is a case study of a 42-year-old woman (who will be called Emma). She had secondary education, was divorced, and raised by herself her 10-year-old daughter and 9-year-old son with autism spectrum disorder. She remained unemployed and used care allowance. Emma came from a very religious family and was raised by mother and grandparents. No one used psychiatric treatment in her mother's family and there was no information about that from the father's side. Her father was born in a Nazi concentration camp and after the war he relied on financial compensation. He was much older than Emma's mother, aggressive, and abused alcohol. He left the family before Emma's first birthday, and reappeared every 6 or 12 months thereafter. According to Emma, her mother was controlling and turned her against her father. Emma also perceived herself as a solution for her mother's pain after miscarrying a child shortly before Emma had been conceived.

Emma had a good relationship with her maternal grandfather, who was supportive and a model of morality and piety. He led her to develop her interest in religion when she was in primary school, but he died when she was 15. Around that time her problems with aggressive or auto-aggressive behavior started, as well as attention seeking by unlawful behavior and breaking school regulations. She became rebellious, and frequently quarreled with her mother who tried to control her social life and sexuality, before running away from home for a year to live in squats, abusing alcohol and drugs, and engaging in risky sexual behavior for money. She was sexually abused under the influence of substance a few times. In adulthood, she lived abroad for almost 10 years, earning money by providing sexual services. During that time she also experienced rape and threats. As she never had close friends, she only used support from priests who, based on her life history, believed she was possessed. She was first exorcized a few times in charismatic groups at age 16. In adulthood, she regularly used deliverance ministries and individual exorcisms according to Roman Catholic ritual for a few years, but her problems and symptoms persisted.

She had elevated levels of somatoform and psychoform dissociation (respectively, measured with SDQ-20 and DESR-PL). More information about her symptoms reported during the clinical interview and mental state examination is provided in Table 3.

**Table 3:** The participant's clinical presentation based on *TADS-I profiles*.

Treatment history	She reports three hospitalizations in the past: first, at age 17, after overdosing drugs and alcohol but being wanted due to having run away from home, she also ran away from the unit; second hospitalization at age 23 and the third one abroad at age 25—both after suicide attempts; no medical records available. She has never used counseling or psychotherapy.
Substance use	*Alcohol*—at age 15 she started to drink beer, wine and vodka; but is unable to define quantity and frequency. Currently drinks recreationally and seldom. *Drugs*—used marihuana every day for a few years from age 17, sometimes used heroin and LSD (especially when living in squats). During her stay abroad, used cocaine several times a month for six months. Currently no drugs. *Medication*—as a teenager, she stole diazepam and other tranquilizers from her mother. At age 17, frequently obtained them on prescription, and mixed them with drugs and alcohol.
Problems with eating	Doesn't report.
Problems with sleep	Doesn't report.
Mood and affect regulation	Her mood fluctuates depending on daily problems (son's school problems and court cases). She has felt depressed and abandoned since ending an intimate relationship with a priest, and been left without support. She has had frequent fantasies of committing suicide by hanging herself on a stole, or stealing the host and putting it into her vagina during intercourse to profane sacred objects. She tends to lose control over sexual or aggressive impulses a few times a month. She maintains this is triggered by prayer and leads to alterations in consciousness. After regaining control she feels ashamed and guilty for what she has done (e.g., sending offensive text messages to her spiritual director).
Fear and panic	She doesn't report clinically significant symptoms. No intrusive memories, avoidance or panic attacks.
Autodestructive behavior	She doesn't report any self-mutilation. Suicide attempts, substance abuse and prostitution in the past. During the episodes of losing control, she sometimes hits the wall.
Self image and identity	She reports many conflicts associated with her sexuality, need for attention, and expressions of anger. She feels guilty for things she has done in the past, contradicting her values and religious beliefs. She also describes herself as strong, stubborn, and reluctant to follow rules. She thinks she is different from other people, spiritually sensitive. She also expresses remorse that she is not as good a mother as she thinks she should be.
Problems in relationships	She reports a great sense of isolation, abandonment and loneliness. She also reveals a great need for attention and being acknowledged. She justifies her tendency for social withdrawal with shame about the work she did abroad. She maintains superficial relationships with people and mainly relies on support offered by clergy. At the same time, she expresses distrust and disappointment in authority figures (teachers, priests). She also feels rejected by the Church after being forbidden to receive the sacrament of penance unless she starts psychiatric treatment. She seeks revenge by using phone or Internet to initiate contacts with men declaring to be priests, exchanging pornographic content, and encouraging them to have sexual conversations. All this proves to her they are dishonest and sinful. She declares having no lay friends and being fully committed to her children.
Problems with sexuality	She denies problems in intimate relationships, although she has been avoiding sexual relations for the last 10 years. During her stay abroad, she offered sex for money, often felt numb and detached from emotions. She also reports having been raped. She feels guilty and ashamed of her past but reports no intrusive memories associated with these incidents. She is afraid of overindulging herself in sex or entering sexual relationships with “the wrong men,” thereby putting her children in danger. Sex-chats with alleged priests evoke in her strong excitement and remorse.
Alterations in consciousness	*Depersonalization*—she frequently felt emotionally detached and numb for short periods of time and without clinical significance. *Derealization*—a sense of being “on a carousel” in stressful moments or during religious activities, leading to aggression.
Somatoform symptoms	She reports “seizures” at home, during which she is unable to move, and trembles, but remains aware of her daughter calling the exorcist for help. She also has convulsions during exorcisms accompanied with rage (biting, kicking, swearing, destroying objects), corresponding to the stereotype of the possession episode—twice a month.
Psychoform symptoms	She does not report amnesia for daily events. She declares some memory gaps for trance episodes at church or events happening when she abused alcohol and drugs. *Schneiderian symptoms*—she has an impression of hearing male voices which encourage her to commit suicide, flirt with priests, or criticize her. Rather than hearing them acoustically, they seem like voiced, intrusive thoughts, which she experiences as ego-dystonic and attributes to “the voice of evil.” She does not report any thought broadcasting or Messianic delusions.
Symptoms indicating a division of self	There is no evidence for the existence of autonomous dissociative parts.
PTSD symptoms	She does not report any.
Summary and diagnosis	She maintains proper orientation, good verbal contact, affect in normal range, denies hallucinations and does not express delusional content, nor provide evidence of it during the interview. She reports episodes of derealization and depersonalization accompanied by partial amnesia limited to changes in behavior and speech, and convulsions. Basic mood and drive within normal limits, proper sleep. She reports problems with self-image and interpersonal relationships which can be interpreted as symptoms of a personality disorder. There is history of suicide attempts but currently does not report suicidal ideations. There were also episodes of using psychoactive substances but she now abstains from them. Changes in behavior and speech with associated alterations of consciousness occur in an isolated manner during religious practices but also at home. This meets the premises for the diagnosis of trance and possession disorders. There are no obvious symptoms of complex dissociative disorders. Her symptoms can be understood in relation to difficult experiences and conflicts about experiencing needs for attention, dependence or unacceptable emotions, such as desire or anger. Her conflicts are additionally reinforced by cultural and religious norms internalized during socialization. F44.3 Trance and possession disorders; Features of personality disorders.

### Data Analysis

Recordings of all the interviews were transcribed verbatim and analyzed together with researchers' notes using qualitative data-analysis software (MaxQDA 2020 ver. 20.4.0). Consecutive IPA procedures were employed in the study (68). Researchers watched each interview and read the transcripts carefully. They individually made notes about body language, facial expressions, the content and language use, reported symptoms, and wrote down their interpretative comments using the annotation feature in MaxQDA 2020. Next, they categorized their notes into emergent themes by allocating descriptive labels. They then compared and discussed their diagnostic insights, coding and interpretations. They analyzed connections between themes and grouped them according to conceptual similarities into main themes.

### Credibility Checks

During each interview, the participant was encouraged to illustrate reported symptoms or experiences with specific examples. Interviewers asked clarification questions to negotiate the meaning the participant wanted to convey. At the end of the interview, she was also asked questions to check that her responses were thorough. The researchers discussed her case thoroughly, including the diagnosis and interpretative notes to compare their understanding of the content and its meaning (the second hermeneutics).

## Results

Emma shared in detail her life history, symptoms and coping strategies. During the follow-up interview (3 years after her assessment), she also described what it meant for her to be diagnosed with possession-trance disorder and how the diagnosis affected her help-seeking. Seven salient themes were identified during the analysis: (1) Excitement and guilt for crossing the taboo, (2) Seeking revenge on priests, (3) Possession-form presentations attracting public attention, (4) The idiom of possession, (5) Exacerbation of destructive behavior during exorcisms makes priests helpless, (6) Making sense of the psychiatric diagnosis, (7) Receiving pastoral counseling as an alternative to professional treatment.

Each theme is discussed and illustrated with verbatim excerpts from the interviews, in accordance with IPA principles.

### Theme 1: Excitement and Guilt for Crossing the Taboo

Emma remembered being introduced to sex when she was six. Secret sex games with her 10-years-older cousin triggered excitement and pleasure, and at first she didn't think of them as harmful. It was only while preparing for her first communion that she learned during confession this was forbidden, shameful and sinful.

It started when I was six. It influenced me because it woke me up too soon and made me sexually licentious later on. I have never talked about it… it was during my confession that I admitted “playing immodestly.” The priest wasn't satisfied and wanted to know the details, he asked who it was with, but I couldn't tell him. I've always taken the blame.

In her secondary school, she repeatedly broke the rules and urged boys to masturbate in class. This excited her and could also make her feel she had control over their sexuality.

I ignored teachers and showed them I did not care. I didn't listen to them at all. I was also so promiscuous. I always sat on the last bench with the boys and forced them to masturbate. I was just very promiscuous.

In her adult life, sexuality became an important arena for inner struggles and conflicts, evoking strong guilt and self-loathing. Emma described sexual desire as “hell,” “the source of corruption” and “mental weakness.” There were periods when she suppressed her needs and abstained from sex and episodes of promiscuous behavior, prostitution, and abusing sexual partners. It seems that eroticism, which she used to regulate her emotions, may have taken the form of a behavioral addiction.

All this sexuality was hell and I would like to spare my children that. Erotica may seem like some kind of beautiful sensory stimulation, but in fact it is losing control over yourself… I was trapped in the claws of erotica, I was so spoiled in that regard. I often met guys who told me that I was hurting them. I just used them only for sex and treated them as objects. I think it was my way of avoiding getting hurt. I would rather take advantage of someone and abandon him, before he could do that to me.

She did seem to have some insight into her problems with attachment and might have used sex to gain control and hide her vulnerability. She thinks this allowed her to avoid emotional involvement with men.

### Theme 2: Seeking Revenge on Priests

In Emma's mind, the Church forbade expressing sexual desire, and commanded abstinence by inducing guilt, shame, and sin. Some priests, like her older cousin when she was a child, both aroused her and were experienced as forbidden targets. She recalled an early exorcism:

These men were holding me face down on the floor. The priest sat on the back of my legs. He kind of lay down on me, holding my hands. He started stroking my hair saying “Accept this love of Christ” and… my body reacted against all reason. I also felt discomfort, because… something happened to me. For the first time I realized I had never thought about a priest before in that way. Looking at a priest, even a handsome one, I had never seen a man, but someone asexual. However, after this exorcism, I had erotic thoughts about him, for 2 weeks. It may have been because I had not been so close to a man for a long time, but common sense won and these emotions subsided. I broke off contact with this exorcist.

When she addressed these moral conflicts with her spiritual father, he first used confession and prayers to help her control sexual impulses. However, she said the relationship evolved into a love affair which made her feel guilty for crossing the taboo. She justified the priest's actions saying it gave her pleasure, which sounds like another repetition of her childhood history.

We became close and finally crossed the physical boundary between a man and a woman. I was not harmed in a physical sense, because it gave me pleasure. It had such an impact on me. Suddenly the whole world, even this whole image of the church, was blown up. I realized I had not overcome my weakness.

In order to deal with conflicting feelings she was seeking men who introduced themselves as priests, engaging them in sex chats and exchanging pornography, subsequently seeing them as equally sinful.

My confessor said we must stop seeing each other, that he was afraid of being alone with me. So I felt really alone. I got really into this virtual reality and started looking for priests who were addicted to erotica. I set up a profile called “Lady for a Priest” and they started contacting me. It was some kind of revenge on my part. Erotica with a normal guy ceased to be attractive—all I wanted was a clergyman. Saturday nights were so exciting, I told them all sorts of things and they masturbated and we exchanged photos. I thought of them celebrating Holy Mass the next day in a state of sin—I felt like a vampire feeding on their weakness, but it also hurt me a lot.

In her mind, Emma seemed to attack and destroy any ideas about the virtues of a person representing authority, morality, or ethics. This could have been a payback for being shamed for her sexuality and for being abandoned. It may also have let her experience moral triumph. However, she also realized that it brought about feelings of loss and pain.

### Theme 3: Possession-Form Presentations Attract Public Attention

Emma learnt from her participation in religious youth camps that unusual behavior which group members ascribed to possession evoked awe and interest and could also lead to receiving special attention for which some adolescents competed.

I got involved in the Oasis movement when I was 15, and was encouraged to participate in a so-called Community Day, a kind of preliminary meeting and excursion. There were three other girls who were acting weird. One started having convulsions while singing. The priests surrounded her… they were standing tightly in a circle, so that no one could see what they were doing. I was really curious and wanted to see what was happening. Since I couldn't break through, I crouched to the side and said: “Satan, if it's your doing, leave her and enter me. I remain at your disposal.” At this point, I lost my temper.

This was the first time Emma's disruptive (violent) behavior was accompanied by derealization and convulsions. She later compared that state to feeling dizzy, as if “riding on a merry-go-round.” Emma remembered being given tranquilizers, which had a delayed effect. She emphasized this was unusual, which for herself and witnesses could have justified the supernatural causation of her state. She saw that youth camp experience as a turning point in her life, leading to further problems, spiritual conflicts, and concentration on religious coping strategies.

It was like being on a merry-go-round, a carousel. My clothes were torn—I fell on stones. The priests went on praying over that girl and after a while she calmed down. I calmed down too and we continued walking, but after a few meters I lost my temper again. It was awful. They called an ambulance and gave me sedatives but it had no effect. A priest said it was a dose for a horse, and it didn't work for me! It was only when the doctor took me to the ambulance away from the priests that I fell asleep so deeply that it was difficult to wake me later. My life has never been normal since.

Since then, in religious contexts Emma regularly experienced bouts of anger, convulsions, and derealization, which subsided when no priest was present. Religious people interpret that as *aversion to the sacred*—clergy see that as one criteria of genuine possession. Her attacks attracted the attention of others and, over time, she engaged in more provocative and socially unacceptable actions. For example, during youth camps she killed and sacrificed a cat. Another time, she had a possession-form presentation after a demonology lecture held at her Catholic high school.

I couldn't resist the temptation to do it, consciously and voluntarily, in front of them… I did a kind of ritual, I grabbed a cat and tore it apart with my hands. Then I offered it to Satan… total madness. Today I am terribly ashamed of it and I can't forgive myself. These girls were horrified because I tore apart a living creature, and most of them came from traumatized backgrounds… Once a priest came to our school and gave a talk about demonology. Me and other girls were preparing the setting and some songs for the holy mass. At one point, as I was going upstairs, I lost it… I started screaming, throwing myself around and hitting myself hard… A girl in my class attempted suicide by taking pills, and then another one. The nuns said it was my influence.

She emphasized that she knew what she was doing and apparently had no amnesia. Despite attributing aggressive impulses to demonic influence, she nevertheless feels guilty and ashamed of her actions. She also realized that she could negatively affect other people, which could also give her a sense of significance and power.

More recently, Emma experienced a strong temptation to steal the host with the aim of profaning it, or fantasized about committing suicide by hanging herself on a priest's stole. These actions shocked and were criticized by the clergy, but were also justified as the manifestations of evil, releasing her from responsibility.

For me, the black mass was the most perfect form of profanation, when the Blessed Sacrament is stolen and placed inside a vagina, preferably just before intercourse. And when the sexual act is done with a priest, it would be the so-called double profanation. I brought home the host and was going to profane it. Not only that… I was planning to commit suicide in a typical satanic ritual of death. I wanted to hang myself on a stole which I had stolen from the church.

Her ideas, which sound as if they were inspired by the *Story of the Eye* by Georges Bataille (72) were popular among people she met in squats, and became banned by the Church. There is no information whether Emma was familiar with such texts, but using the expression “so-called double profanation” suggests she shared common knowledge about the libertine movement.

### Theme 4: The Idiom of Possession

Emma has extensively studied literature about demonology and watched local exorcists preaching on YouTube about “spiritual threats” and “spiritual warfare”. This has made her believe that premature or unwanted sexual activity, interest in occultism, exposure to foreign symbols, philosophies and treatments (e.g., magnetic healing) can make one prone to demonic possession.

When I was a child, the first bad thing that happened to me was the attack on my innocence, sexual I mean. That evil was silent, unnoticeable. Later, my grandmother took me to a magnetic healer, which only made things worse. You know, there is bad energy which can be transferred, because God does not bestow such healing powers. If he heals, it only happens through sacraments and prayer. Later, as a child, I had trouble praying. Every time I knelt down to pray, I felt that my prayer was wrong. Something prevented me from finishing it, so I had to get up and start over. These are things that led to this evil. And then it just got worse and worse when I got involved in drugs.

She also believed in so-called “manifestations” associated with the “aversion to sacrum.” These possession-form presentations are regarded as indicators of possession in a theological sense, and are often discussed by religious community members. Emma observed that since her first “possession” experience at the Oasis gathering (see: Theme 3), she grows agitated, angry, and gets convulsions during prayers. On the other hand, all her symptoms subside when she is not involved in religious matters. This only reinforced her belief that she is truly possessed.

I was recently playing with my kids at home and suddenly I started shaking. This merry-go-round appears and I bounce off the wall like a ball. I later found out that when this happened the priest was praying for me (my daughter knows him and called him for help). If he prays, it only prolongs everything. If he does not pray, my children call me back, they shout “mommy, mommy, mommy,” then I follow their voices and quickly return to my senses. The worst thing was when my daughter turned on the speakerphone and I could hear that priest praying. I had asked him never to do that when I was at home with the children, so that they are not threatened. It is not about me, but about the children. I don't want them to witness that. Unfortunately the priest… I guess, my daughter felt safe because he was praying. Maybe she felt she wasn't alone, but it only prolonged everything.

This reveals interesting interactions between Emma, her children and the exorcist. She seemed to experience conflicts about receiving attention and help. Her daughter sought refuge in the fatherly figure but, according to Emma, this only escalated her symptoms. She partially identified with the feelings of helplessness and abandonment of the 10-year-old girl, but also saw herself as a potential source of threat for her children.

During the interview, she reported strong temptations to do unacceptable things, which she ascribed to “inner voices” but denied having auditory hallucinations *per se*. Her “voices” were thought-like experiences and represented good and evil. Sometimes, she engaged in inner dialogues with them.

I have three voices inside and sometimes I talk to them. There is the good side which usually speaks softly, calms me down, and develops some sense of peace. The second voice usually exerts pressure. And there is my voice, that of my psyche, which questions the previous one and tries to make sense of it all.

Rather than perceiving them as an expression of her own mental activity, she attributed these thought-like voices to the supernatural, saying only individuals who believe in God could understand the spiritual dimension of such phenomena. She emphasized that they need to resolve conflicts between their instincts and moral values. The struggle between good and evil is not merely symbolic in her narrative, but attributed to concrete entities.

In order to understand these voices, one must acknowledge the existence of God because, if we reject the existence of God, the existence of these voices becomes irrational. Every Christian has this dimension of the struggle in his conscience. There is a good side, and there is a bad side.

Emma had endorsed and identified with the concept of possession and navigated between its clinical and theological meanings. Despite multiple and ineffective exorcisms, she seemed reluctant to accept priests' suggestions that she might have a psychological disorder. Even a clinical diagnosis, according to her, did not rule out spiritual causation of problems.

Even if someone is mentally ill, it does not mean that no devil's work is involved here, because the devil can work through disease. Satan wants to drive as many people as possible away from God. He will take advantage of every disease, every weakness or flaw which he can use to do evil.

She maintained that genuine spiritual possessions are uncommon but do occur and prove to the faithful the existence of the supernatural.

I think possession, from this theological point of view, is something which rarely or practically never happens. And when it does happen, it's like a sign from God to confirm that this spiritual world exists.

### Theme 5: Exacerbation of Destructive Behavior During Exorcisms Makes Priests Helpless

First attempts to expel evil spirits from Emma began when she was 15. She took part in deliverance ministries organized in a Christian group where the pastor and his male assistants took her aside and tried to tame her. Her agitation and resistance was interpreted as a sign of possession. Emma managed to escape one such event and, when news about this spread, the local parson and her mother forbade her from joining that group again.

They picked me up a few times and took me to the church. In this Christian community, a group of men—the pastor and his assistants—took me to a separate room and simply tried to force the evil spirit out of me. When I resisted, they used physical force… they tied my hands, they sat on my legs, someone sat on my arm. They hit me and used gestures to chase away the evil. It was like in American films. I felt they were crossing my boundaries but when I tried to protest they saw it as the manifestation of evil. It was painful and I was bruised and torn. I broke free once and ran away, leaving my things behind. I went to the parish and asked for help. The parish priest and my mother were told about everything and they insisted I never go there again.

Because her behavior was inappropriate and scandalous in high school, nuns who ran the school sent her to an exorcist. During this and subsequent rituals she grew more and more violent, offended priests, damaged chapels, and became even more furious when they tried to restrain her.

After leaving hospital [after a suicide attempt], I sought help in the parish. One priest arranged a meeting for me at the church with three other priests who wanted to pray with me, but I just… just demolished the church. I smashed figures, windows, and broke benches. They were unable to do anything and even called the police. The priest said he couldn't help and I must be taken to an exorcist.

Being labeled as “possessed” legitimized Emma's aggressive behavior toward authority and allowed her to feel she had special spiritual significance. It was also the source of secondary gains: she received special attention and emotional support from clergy. Perhaps this is why she was reluctant to give up on the priests, even when there was no improvement and they felt helpless against her behavior. Over time, she consulted different exorcists who subjected her to exorcism, until they questioned her possession and insisted on a psychiatric consultation.

I visited the Pauline order for the deliverance ministry and was flailing around all day. After praying over me for a long time they gave up and said it might be a mental illness because the prayers weren't working. They hoped I would change and calm down under their influence but I was flailing as long as they were praying. Finally, they came to the conclusion that I must be mentally ill and the Church could not help.

### Theme 6: Making Sense of the Psychiatric Diagnosis

Emma obediently went for a diagnostic appointment as if on her own initiative. She revealed some disappointment with exorcists who had tried to help her. She saw them as helpless and confused, but also ascribed a certain level of arrogance to them.

I would like someone to look at my experiences from a different, specialized perspective, unlike the one provided by priests. There are many exorcists in Poland but these priests are very confused, although they think they are wise and know best.

She seemed certain in her beliefs about demonic agency and was reluctant to consider alternative explanations for her aggressive and sexual impulses. Despite receiving psychoeducation about emotional regulations and the meaning of PTD, she felt that by using the term “possession” clinicians only supported her theories.

In the follow-up interview 3 years later, Emma could not hide her disappointment that the priests had lost interest in her case. It seems that receiving a medical diagnosis was an excuse for them to stop further exorcisms and deny her sacraments unless she started psychiatric treatment. Paradoxically, Emma said this proved their fear and helplessness about her being genuinely possessed, which she thinks was only confirmed by mental health specialists.

This diagnosis is not so clear… In people from outside, it evokes… because if something is not clearly specified, indicated in writing, then you may still hesitate and have different theories. When they have confirmation that they are dealing with something… For example, when I gave this [report] to the confessor [takes a loud breath], when he read “trance and possession,” for him there was no medical dimension, he wasn't thinking in medical or psychological terms, but theological. In theological terms, there is some force, a force that is beyond your powers… I came to the conclusion that before I had this [PTD] diagnosis, they kept trying to help me and willingly controlled my life. But when they realized I was possessed, they just stopped because it was too overwhelming for them… They completely rejected me, avoided any kind of services, confession.

Subsequently, she also broke her contacts with charismatic groups and moved to another town. She still celebrated holy mass every Sunday but withdrew from all groups and focused on helping her children adapt to a new environment. Her convulsions and derealization (feeling of merry-go-round) disappeared.

### Theme 7: Receiving Pastoral Counseling as an Alternative to Professional Treatment

Although being denied further exorcisms caused Emma to leave the charismatic groups, she seemed reluctant to use the professional treatment which was recommended to her after the diagnostic consultation. She professed faith in providence, saying mental health professionals could potentially express God's grace, but never pursued any therapy.

Divine providence shows us the way forward, which can even be in the form of therapeutic help. God does not work in a supernatural way but through everyday life, also through lay people rather than priests.

Perhaps this was her way for rebelling against the priests who told her that treatment was a condition *sine qua non* for receiving the sacraments. Instead, she moved to a new town and completely changed her environment. She also found a compromise solution in the form of pastoral counseling. Emma said she liked her new spiritual director and accepted his strict rules regarding appointments and timing.

I met a young priest in the confessional. I asked him for so-called spiritual direction and he agreed. I felt obliged to show him this [diagnostic] report and he wasn't scared [that I was possessed]. I was surprised by his maturity and willingness to serve other people. This priest… he treats me completely differently. He sticks to our arrangements, which is very cool. For example, as time passes and we are about to end… we start punctually at 4:00 p.m. and as 4:45 is approaching, he nicely sums up what we have talked about. He teaches me about boundaries.

## Discussion

Emma has a history of problems relating to attachment, emotional regulation and self-image indicating features of a personality disorder. She also reported somatoform symptoms accompanied by alterations in consciousness. Her ‘attacks' were not only limited to religious rituals or other situations associated with the church but also occurred outside the religious context. For this reason, PTD was an adequate diagnosis, despite her evident personality problems.

People reporting possession are a heterogenous group from the diagnostic perspective. Clinicians need to consider conditions other than PTD potentially valid categories, in particular: personality disorders (13), complex dissociative disorders or PTSD (20, 21, 54, 73) or psychosis (14). Analyzing stressful experiences, existing psychological conflicts and the pathways to the disorder can shed light on mechanisms behind alterations of consciousness and behavior, and potential gains from illness (4, 31). This was possible to see in our case study.

Exploring the complexity of ego-dystonic behavior may also reveal if possession-form presentations merely express conflicting and disowned emotions and needs, or reflect a more complex dissociative structure. In the former case, endorsement and identification with the possession can help people justify their shameful aggressive or sexual acts. In a similar way, people with false-positive DID use the learnt concept of alternative identities or parts to receive attention or express conflicting emotions (74). In a more fragmented psyche, however, the possessing agent can embody an autonomous dissociative part with a first-person perspective. Their degree of mental autonomy and complexity may differ—from simple ego-dystonic parts embodying particular ego states (e.g., rage), to fairly complex, characterful parts with their own sense of identity, motives, and memories.

According to ICD-11, the boundary between PTD and DID lies in the attribution of the possessing agent: external in PTD and not external in DID/Partial DID. There seems to be no scientific evidence for that distinction. Moreover, clinical observations indicate that DID patients experience their alternate identities as alien and ego-dystonic, therefore the external / internal attribution is not very clear in them (75).

We also share doubts about the validity of PTD. Its diagnostic criteria include both symptoms (changes in behavior and sense of identity accompanied by alterations in consciousness) and meaning which patients attribute to them. This way of formulating diagnostic criteria would rather justify treating PTD as a culture-bound syndrome. Whereas, culture shapes the way in which people express and interpret their symptoms, and possession by the goddess Kali, devil, or evil spirits in a Buddhist context may present itself differently, Ross et al. (53) postulate that there is an underlying dissociative pathology in people with culture-bound syndromes which should be carefully examined. This means that in making a diagnosis clinicians should focus on symptoms rather than ascribed meaning.

Further research is also necessary to assess the usefulness of a PTD diagnosis, as this category can have serious implications for patients, their families and spiritual community, and even healthcare providers. In those who use religious services, it can reinforce the belief in the supernatural causation of their symptoms, make them reluctant to use recommended treatment and overemphasize religious coping. Externalization of conflicts does not necessarily promote seeing and embracing unaccepted impulses as one's own. While this may be functional, it may block further development and be iatrogenic (13). Furthermore, the double meaning of “possession” (as a cultural and medical concept) makes it difficult for some specialists to think about their patients in strictly clinical terms. Subsequently, they consider “genuine possession” as a possible explanation for reported symptoms (12, 25, 26). Because of the religious connotations, we recommend to reconsider the name and diagnostic criteria of PTD. It might be more appropriate to use the 6B6Y category in ICD-11 (Other specified dissociative disorders) for people with this clinical presentation, similarly to DSM-5.

Feeling trapped between the cultural and medical notions of possession can influence the way mental health professionals collaborate with clergy in diagnosing and treating patients with possession-form presentations. For some, it may be difficult to maintain clear professional boundaries: identify and describe symptoms of clinical significance, formulate accurate diagnosis, and offer psychoeducation and treatment, at the same time, staying culturally sensitive and understanding the need for spiritual support sought by patients. The role of the clinician is not to exclude the spiritual causation of reported symptoms but to offer an alternative understanding of psychological conflicts and unmet needs.

## Limitations and Further Directions

IPA studies, being focused on how people experience phenomena and meaning-making, are naturally limited to small samples or even case studies. Care should be taken in drawing conclusions from such qualitative studies and further research using rigorous methodology is required to explore problems and illness behavior in people with possession-form presentations. More studies are necessary comparing PTD with other dissociative disorders (Partial DID and DID), analyzing in particular different clusters of symptoms and emotional regulation. Longitudinal studies exploring the development of symptoms and help-seeking pathways, and meaning attributed to the medical and folk diagnoses are also recommended.

## Conclusions

Our review of literature shows that PTD requires further scientific evidence to remain a valid clinical diagnosis. Thorough diagnostic assessments should be applied to people reporting possession-form presentations, not only exploring their phenomenological features, but also accompanying problems and symptoms. Use of the term “possession” by mental health professionals is also burdened with social consequences. It can reinforce patients' beliefs about the supernatural causation of problems and affect help-seeking. We thus stipulate that the “Other” specified dissociative disorders category in ICD-11 could be more appropriate for people with this clinical presentation.

## Data Availability Statement

The datasets presented in this article are not readily available because Polish law regarding medical history and the guidelines of the Ethical Board do not allow distributing the transcripts of the interview or psychiatric assessment. This material contains patient's identifiable information. Furthermore, transcripts include about 70 pages of text in Polish which cannot be translated and edited to mask patient's details. Requests to access the datasets should be directed to ipietkiewicz@swps.edu.pl.

## Ethics Statement

The studies involving human participants were reviewed and approved by Ethical Review Board at the SWPS University of Social Sciences and Humanities. The patients/participants provided their written informed consent to participate in this study. Written informed consent was obtained from the individual(s) for the publication of any potentially identifiable images or data included in this article.

## Author Contributions

IJP developed the concept of this research, collected clinical data, analyzed literature and transcripts, and wrote manuscript. UK transcribed interviews and helped in literature review and data analysis. RT performed psychiatric assessment, participated in analyzing interviews, and reviewed the manuscript. All authors contributed to the article and approved the submitted version.

## Funding

This work was supported by a research grant from the National Science Centre, Poland: 2017/25/B/HS6/01025.

## Conflict of Interest

The authors declare that the research was conducted in the absence of any commercial or financial relationships that could be construed as a potential conflict of interest.

## Publisher's Note

All claims expressed in this article are solely those of the authors and do not necessarily represent those of their affiliated organizations, or those of the publisher, the editors and the reviewers. Any product that may be evaluated in this article, or claim that may be made by its manufacturer, is not guaranteed or endorsed by the publisher.
